# Mitigation of Mycotoxin Content by a Single-Screw Extruder in Triticale (*x Triticosecale* Wittmack)

**DOI:** 10.3390/foods14020263

**Published:** 2025-01-15

**Authors:** Breda Jakovac-Strajn, Janja Babič, Lato Pezo, Vojislav Banjac, Radmilo Čolović, Jovana Kos, Jelena Miljanić, Elizabet Janić Hajnal

**Affiliations:** 1 Institute of Food Safety, Feed and Environment, Veterinary Faculty, University of Ljubljana, Gerbičeva 60, 1000 Ljubljana, Slovenia; janja.babic@vf.uni-lj.si; 2 Institute of General and Physical Chemistry, University of Belgrade, Studentski Trg 12–16, 11000 Belgrade, Serbia; latopezo@yahoo.co.uk; 3 Institute of Food Technology, University of Novi Sad, Bulevar Cara Lazara 1, 21000 Novi Sad, Serbia; vojislav.banjac@fins.uns.ac.rs (V.B.); radmilo.colovic@gmail.com (R.Č.); jovana.kos@fins.uns.ac.rs (J.K.); jelena.miljanic@fins.uns.ac.rs (J.M.); elizabet.janich@fins.uns.ac.rs (E.J.H.)

**Keywords:** extrusion processing, whole-grain triticale flour, deoxynivalenol, 3-Acetyldeoxynivalenol, 15-Acetyl-deoxynivalenol, HT-2 toxin, tentoxin, alternariol monomethyl ether, mycotoxins reduction, UPLC-MS/MS

## Abstract

The aim of this study was to investigate the effects of extrusion processing parameters—moisture content (*M* = 20 and 24%), feeding rate (*FR* = 20 and 25 kg/h), and screw speed (*SS* = 300, 390 and 480 RPM), on the content of deoxynivalenol (DON), 15-Acetyl Deoxynivalenol (15-AcDON), 3-Acetyl Deoxynivalenol (3-AcDON), HT-2 Toxin (HT-2), tentoxin (TEN) and alternariol monomethyl ether (AME), using a pilot single-screw extruder in whole-grain triticale flour. The temperature at the end plate of the extruder ranged between 97.6 and 141 °C, the absolute pressure was from 0.10 to 0.42 MPa, the mean retention time of material in the barrel was between 16 and 35 s, and the specific energy consumption was from 91.5 to 186.6 Wh/kg. According to the standard score, the optimum parameters for the reduction of the content of analysed mycotoxins were *M* = 24 g/100 g, *FR* = 25 kg/h, *SS* = 480 RPM, with a reduction of 3.80, 60.7, 61.5, 86.5, 47.7, and 55.9% for DON, 3-AcDON, 15-AcDON, HT-2, TEN, and AME, respectively. Under these conditions, the bulk density, pellet hardness, water absorption index, and water solubility index of the pellet were 0.352 g/mL, 13.7 kg, 8.96 g/g, and 14.9 g/100 g, respectively.

## 1. Introduction

Mycotoxins are secondary metabolites produced by several genera of filamentous fungi, including *Aspergillus*, *Fusarium*, *Penicillium* and *Alternaria* [1,2,3]. These mycotoxins pose a significant health risk to humans and animals [1,2,3]. Given the serious threat of mycotoxins present in the food chain, it is essential to effectively remove or reduce their concentrations in food. The most common mycotoxins produced by *Fusarium* species (*F. sporotrichioides*, *F. graminearum*, and *F. verticillioides*) are deoxynivalenol (DON), its acetylated metabolites 3-acetyl DON (3-AcDON) and 15-acetyl DON (15-AcDON), zearalenone (ZEA), fumonisins, T-2 and HT-2 toxin, and diacetoxyscirpenol [2,4,5]. *Fusarium* moulds mainly infect grains such as wheat, maize, and rice, while *Alternaria* moulds affect various plants (vegetables, fruits) and cereals. The most important mycotoxins produced by *Alternaria* spp. are alternariol (AOH), alternariol monomethyl ether (AME), tentoxin (TEN), and tenuazonic acid (TeA) [6,7,8,9].

Exposure to one or more mycotoxins usually occurs through the ingestion of contaminated feed or food. The accumulation of *Fusarium* mycotoxins in the body can lead to various acute or chronic effects, including teratogenic, neurotoxic, embryotoxic, and immunosuppressive effects [1,10,11]. Unfortunately, toxicological data for *Alternaria* toxins are limited and incomplete, as neither bioavailability studies nor long-term clinical studies are available [8,9]. However, exposure to *Alternaria* toxins is known to cause cytotoxic, fetotoxic, and teratogenic effects in animals and humans, which have been associated with a range of pathologies, from haematological disorders to oesophageal cancer [8,9].

Various physical (sorting, sieving, cleaning, flotation and density separation, washing, milling, heat treatment, extrusion, soaking, irradiation, cold plasma, mycotoxin binder, temperature and humidity control during storage, modified atmosphere treatment, and irradiation treatment), chemical (chemical antifungal agents, photodynamic treatment, electrolytic oxidation water treatment, plasma treatment), and biological (inhibition by microorganisms and their metabolites, inhibition by plant extracts) methods are used in industrial and laboratory food processing to control fungal growth and prevent mycotoxin formation [7,12,13,14,15]. Although these methods can reduce mycotoxin contamination to safe levels, they often lead to significant changes in the food substrate, such as changes in colour, taste, lipid oxidation and vitamin degradation [7,12,13,15]. Thermal treatments such as cooking, boiling, frying, roasting, canning, baking, and extrusion can also reduce mycotoxin contamination. Among these heat treatments, extrusion has great potential to reduce mycotoxins due to the combination of high temperature, high pressure, and shear forces, as most mycotoxins are chemically and thermally stable at temperatures close to 100 °C [7,15]. The effectiveness of extrusion in reducing mycotoxins depends on factors such as the type of mycotoxin, the addition of food additives, and the extrusion conditions (e.g., temperature, moisture, screw type, screw speed, pressure, and residence time in the extruder) [5,7,15]. The extruders used for this process can be either single-screw or twin-screw extruders. In single-screw extruders, a single screw rotates in a barrel and moves the raw materials through various processing stages. A key advantage of single-screw extruders is their ability to efficiently process different types of raw materials, including grains and other plant materials [7,16]. Pellet production with a single-screw extruder is an efficient process for converting grain and other raw materials into a homogeneous and stable form suitable for animal feed or human consumption [7,16].

The effectiveness of extrusion in reducing various mycotoxins in wheat, maize, barley, oats, and rice has been well documented [5,15,16,17,18,19,20]. In 2016, Janić Hajnal and colleagues investigated the potential for reducing *Alternaria* toxins in whole wheat flour using a single-screw extruder [21]. The same research group later investigated the effect of twin-screw extrusion on *Fusarium* and *Alternaria* mycotoxins in whole triticale flour (2022) [22] and the reduction of *Alternaria* toxins in whole-grain red sorghum flour (2024) [23]. This study aims to investigate the extrusion process using a single-screw extruder as an effective method for reducing various *Fusarium* (DON, 3-AcDON, 15-AcDON, HT-2) and *Alternaria* mycotoxins (TEN, AME) in whole-grain triticale flour. Modern mathematical techniques, such as Response Surface Methodology (RSM) highlighted by Grasso [24], offer methods for optimising the extrusion process. These techniques allow the regulation of extrudate quality while simultaneously evaluating the influence of extrusion parameters on mycotoxin reduction.

The EU Commission Regulations No. 2023/915 [25] and No. 2024/1038 [26] specify the maximum permitted levels for DON and the sum of T-2 and HT-2 toxins in unprocessed cereals and various bakery products, cereals and snacks. Specifically, the maximum level for DON in unprocessed cereal grains is limited to 1250 µg/kg, while it is set at 500 µg/kg for bread, pastries, biscuits, cereal snacks and breakfast cereals [25]. For the sum of T-2 and HT-2 toxins, the maximum level in unprocessed cereal grains is limited to 50 µg/kg, while it is set at 20 µg/kg for baked goods, pasta, cereal snacks and breakfast cereals [26]. Although the maximum levels for common *Alternaria* toxins (AOH, AME and TeA) are not yet regulated, the EU Commission issued a recommendation (No. 2022/553) in 2022 to monitor the presence of these toxins in food [27]. The guideline values for AOH (2 µg/kg), AME (2 µg/kg) and TeA (500 µg/kg) in certain foods suggest that if these values are exceeded, investigations should be carried out to determine the factors contributing to the presence of *Alternaria* toxins and the effects of food processing. It should be noted that guidance values are not food safety values [27].

In this study, we aim to optimise the extrusion procedure using whole-grain triticale flour. In particular, we will investigate how changes in extrusion parameters—screw speed, feeding rate, and material moisture content in the extruder barrel—affect both the reduction of *Fusarium* and *Alternaria* toxins and the quality of the resulting pellets. With an optimised extrusion process as a modern technological solution to reduce mycotoxins, the food industry can ensure safer and higher-quality food for the end consumer. 

## 2. Materials and Methods

### 2.1. Material Grinding and Mixing

Triticale grain (300 kg) was provided by the Institute of Field and Vegetable Crops, Novi Sad (Serbia). The grain was ground to a fine powder using a hammer mill (model 9FQ-50, XT Machinery, Shanghai, China) powered by a 22 kW electric motor. This mill had 16 hammers arranged in four rows and a sieve with a diameter of 1 mm. The resulting powder was then homogenised for 90 s in a Muyang SLHSJ0.2A double-shaft paddle mixer (Muyang, Yangzhou, China). The mixing homogeneity of triticale flour was confirmed through the Microtracer^®^ method, in which external tracers are used to assess mixing homogeneity [28]. In addition, twelve subsamples were taken to analyse the mycotoxin levels in naturally contaminated whole-grain triticale flour.

### 2.2. Extrusion Conditions

**Figure 1:**
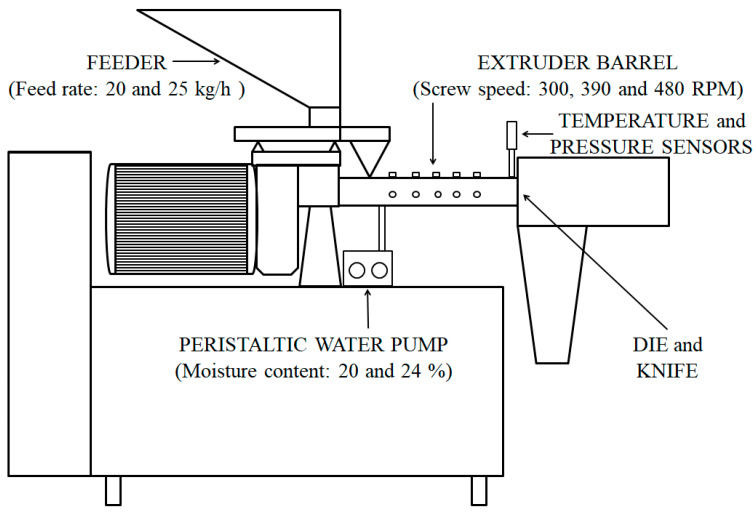
Scheme of the single screw extruder.

The triticale pellets were produced using a single screw extruder (model OEE 8, Amandus Kahl GmbH & Co. KG, Reinbek, Germany). The scheme of the single screw extruder is presented in Figure 1. This type of extruder had no jacketed heating or cooling system, so the heat was generated only by the friction between the material, the screw and the inner walls of the barrel. The extruder was fed via a volumetric screw feeder, while the water was added to the feeding section of the barrel using a peristaltic pump Watson Marlow 400 (Watson Marlow, Cornwall, England). The extruder die had two round-shaped openings with a diameter of 5 mm (total opening area of 39.25 mm^2^) while the rotary knife with two blades was used to achieve the final length of the product. The temperature of the material in the barrel (*T*) was measured with a Pt100 probe that was positioned at the end section of the barrel. The pressure at the die (*PEP*) was monitored by a sensor right before the material entered the die. The value of energy consumption value during extrusion was read out from the control display of the device and was used for determining specific energy consumption (*SEC*). Screw speed (*SS*) was varied at three levels (300, 390 and 480 RPM), while feeding rate (*FR*) and moisture content of the material inside the barrel (*M*) were varied at two levels (*FR*: 20 and 25 kg/h; *M*: 20 and 24%). Twelve samples (SS-1 to SS-12) in the form of pellets were produced in total. The mean retention time (*t*) of the material in the extruder barrel was determined by introducing the coloured tracer particles in the inlet of the barrel together with the material and measuring the time needed for the coloured material to exit the barrel. Drying and subsequent cooling of the extrudates were performed in a fluidised bed vibro dryer/cooler (model FB 500 × 2000, Amandus Kahl GmbH & Co. KG, Reinbek, Germany).

### 2.3. Physical and Chemical Analysis

The moisture content was determined in both the triticale flour and the extruded products by the established ISO 712/2009 standard method [29]. The bulk density (*BD*) of each pellet sample was measured in triplicate with a bulk density tester (Tonindustrie, West und Goslar, Germany). The pellet hardness (*PH*) was determined by Texture Analyser (model TA.HDPlus, Stable Micro Systems Ltd., Godalming, UK) fitted with a 50 kg load cell. The following test settings were used: pretest speed: 2.0 mm/s; test speed: 0.16 mm/s; post-test speed: 10 mm/s; distance: 2.5 mm; trigger force: 100 g. The force–time graph’s maximum peak force was considered an indicator of hardness, and it was expressed in kg as the mean of the results of 20 extrudates from each trial. The water absorption index (*WAI*) and the water solubility index (*WSI*) of pellets were determined by Anderson et al. [30] with minor modifications. Briefly, in a weighted 15 mL glass centrifuge tube, 0.2 g of ground extrudates were suspended in 5 mL of distilled water. After two minutes of stirring on a Vortex mixer (VELP Scientifica Srl - HQ, Usmate, Italy), the tube was centrifuged at 5000× *g* for twenty minutes at room temperature (25 °C) using an Eppendorf Centrifuge 5804 R, Hamburg, Germany. An evaporating dish with a known weight was used to decant the supernatant. After the supernatant was decanted, the gel was measured for the *WAI* calculation. The *WSI* was determined from the weight of dry solids after evaporation of supernatant from the *WAI* test at 105 °C in a drying oven (model UNB 400, Memmert GmbH + Co. KG, Schwabach, Germany). The *WAI* and *WSI* were calculated according to the equations given in the publication by Janić Hajnal et al. [22].

### 2.4. Mycotoxin Determination by UPLC-MS/MS Analysis

Sample preparation and quantification of the analysed *Fusarium* and *Alternaria* toxins (DON, 3-AcDON, 15-AcDON, HT-2, AME, and TEN) in whole grain triticale flour and pellets was performed using ultra-performance liquid chromatography coupled with a triple-quadrupole mass spectrometer (UPLC-MS/MS; Waters, Milford, MA, USA). We used the same sample preparation protocol, analytical equipment, chemicals, reagents and materials as Topi et al. [31] and Babic et al. [32]. Ten grams of ground cereal sample was extracted with acetonitrile:deionised water (84:16, *v*/*v*) by shaking for 1 h. The extract was filtered, and a four ml aliquot of the sample was dried at 60 °C under vacuum. The sample was redissolved in methanol:deionised water (20:80, *v*/*v*). The dissolved solution of the sample was injected into the UPLC-MS/MS system. The injection volume was 7.5 µL. To separate the mycotoxins, gradient chromatography was performed with mobile phases A (water) and B (methanol), each with the addition of 2.5 mM ammonium acetate and 0.5% acetic acid. All mycotoxins were determined with the MS/MS detector in ESI +/− mode. LC–MS/MS parameters for the analysed mycotoxins are described by Topi et al. [31] and Babič et al. [32]. Mycotoxins before and after extrusion processing of whole-grain triticale flour were quantified using previously validated and published methods without any modifications [22].

### 2.5. Statistical Analysis

The data were statistically analysed using the TIBCO Statistica^®^ 14.0.0.15 software [33] software package (StatSoft Inc., Tulsa, OK, USA). All analyses were carried out in triplicate, with the results given as mean values with standard deviations (SD). The equality of variance between samples was tested using Levene’s test, while normality was assessed using the Shapiro–Wilk test. Analysis of variance (ANOVA) was applied to analyse the differences between the twelve triticale samples in terms of die temperature; pressure at the die; specific energy consumption; mean retention time in the barrel; bulk density; pellet hardness; water absorption index; water solubility index; reduction of mycotoxins (deoxynivalenol, 3-acetyl deoxynivalenol, 15-acetyl deoxynivalenol, HT-2 toxin, tentoxin, alternariol monomethyl ether).

#### 2.5.1. Principal Component Analysis

The inter-correlation between variables was analysed by calculating the correlation matrix and examining its significance using SPSS software, ver. 25. To ensure the accurate interpretation of factors, varimax rotation was performed in the factor analysis. The Kaiser–Meyer–Olkin test (KMO) was used to test sample adequacy [34] and the Bartlett test for sphericity [35] was used to test suitability for factor analysis.

Principal Component Analysis (PCA) was used to recognise and identify patterns within the collected data. In addition, an analysis of variance (ANOVA) was performed to assess the influence of the factor variables on the responses.

#### 2.5.2. Response Surface Methodology

The extrusion process of whole grain triticale flour was influenced by three-factor variables: screw speed—*SS* (300, 390, and 480 RPM), feeding rate—*FR* (20 and 25 kg/h), and moisture content *M* (20 and 24%). These factor ranges were determined based on the basis of preliminary tests. Using an experimental design with three levels for each of the three factors, the collected experimental data were analysed to evaluate the effects. With a limited sample size of 12, this proved sufficient for evaluating second-order polynomial (SOP) coefficients [36].

#### 2.5.3. Standard Score

Standard scores were determined for various mycotoxin reduction trials using the extrusion method. The ranking procedure was based on the ratio between the raw data and the extreme values for each response [22].

## 3. Results

### 3.1. Determination of Examined Mycotoxin Content

To determine the *Fusarium* and *Alternaria* toxins studied, an external matrix-matched calibration approach was utilised to mitigate potential matrix effects. Both the extruded product (pellet) samples and the whole-grain triticale flour underwent separate calibrations. The resulting data were adjusted for recovery (R) and expressed based on dry matter content. On a dry weight basis, the initial water content of the naturally contaminated whole-grain triticale flour was 10.9 g/100 g, while the water content of the 12 sets of pellets ranged from 8.1 to 13.5 g/100 g. The initial concentrations of the quantified mycotoxins in whole-grain triticale flour were 274.4 ± 36.4 μg/kg for DON, 2.86 ± 0.24 μg/kg for 3-AcDON, 4.86 ± 0.43 μg/kg for 15-AcDON, 4.59 ± 0.42 μg/kg for HT-2, 29.8 ± 1.78 μg/kg for TEN, and 16.7 ± 5.37 μg/kg for AME (averages of twelve measurements). Each set of pellets (extruded products) was analysed twice. The reduction of analysed *Fusarium* and *Alternaria* toxins by extrusion processing is presented in Table 1.

### 3.2. Reduction of Examined Mycotoxins via Single-Screw Extruder

It was found that screw speed (*SS*), feeding rate (*FR*), and moisture content (*M*) impact the observed responses (DON, 3-AcDON, 15-AcDON, HT-2, AME, and TEN reduction rate, *T*, *PEP*, *SEC*, *t*, *BD*, *PH*, *WAI*, and *WSI*) in the extrusion process (Table 1).

ANOVA analysis was performed to evaluate the differences in content between samples. The *p*-value indicated the statistical significance of differences between samples (at the *p* < 0.05 level), while the percentages represented the relative changes in mycotoxin content between conditions. All statistical analyses performed examined the reduction (expressed as a percentage reduction relative to the initial concentration in whole-grain triticale flour) of *Fusarium* and *Alternaria* toxins during the extrusion process.

The responses of the pilot single-screw extruder varied in the following ranges: temperature (*T*) from 97.6 to 141 °C, pressure (*PEP*) from 0.10 to 0.42 MPa, specific mechanical energy (*SEC*) from 91.5 to 186.6 kWh/t, and mean retention time in the barrel (*t*) from 16 to 35 s. The content of every *Fusarium* and *Alternaria* toxin decreased during the extrusion process (Table 1). The reduction of DON ranged from 0.72 to 7.27%, while the reductions for 3-AcDON, 15-AcDON, and HT-2 ranged from 0.26 to 60.7%, 7.13 to 86.5%, and 17.3 to 86.5%, respectively. The reduction of examined *Alternaria* toxins during the extrusion process ranged from 1.77 to 47.7% for TEN and 10.6 to 55.9% for AME.

As shown in Table 1, the maximum reduction of most of the quantified mycotoxins was achieved during the extrusion of whole-grain triticale flour using the same process parameters. The maximum reduction rates for 3-AcDON (60.7%), HT-2 toxin (86.5%), TEN (47.7%), and AME (55.9%) were achieved at *M* = 24%, *FR* = 25 kg/h, and *SS* = 480 RPM (SS-12). Conversely, the most significant reduction rate for DON (7.27%, SS-6) was obtained at the lowest moisture content in the extruder barrel (20%), the highest feeding rate (25 kg/h), and the highest screw speed (480 RPM). The highest reduction rate for 15-AcDON (66.7%) was achieved at *M* = 20 kg/h, *FR* = 20 kg/h, and *SS* = 480 RPM (SS-2). In addition, the physicochemical quality indicators of the obtained pellets, were in the following range depending on the applied process parameters: *BD* from 0.183 to 0.418 g/mL, *PH* from 9.08 to 18.4 kg, *WAI* from 4.66 to 8.98 g/g, and *WSI* from 12.6 to 38.5 g/100 g.

Following the main objective of this investigation, the results of applying contemporary mathematical techniques are presented below.

### 3.3. Principal Component Analysis

Factor loadings below 0.3 were suppressed, and the assumption of positive definiteness was fulfilled, as indicated by a determinant value greater than zero (3.570 × 10^−10^). The calculated KMO value of 0.930 confirmed a strong interdependence between the variables, and Bartlett’s test revealed significant sphericity (*p* < 0.001) so that Principal Component Analysis could be performed. 

**Figure 2:**
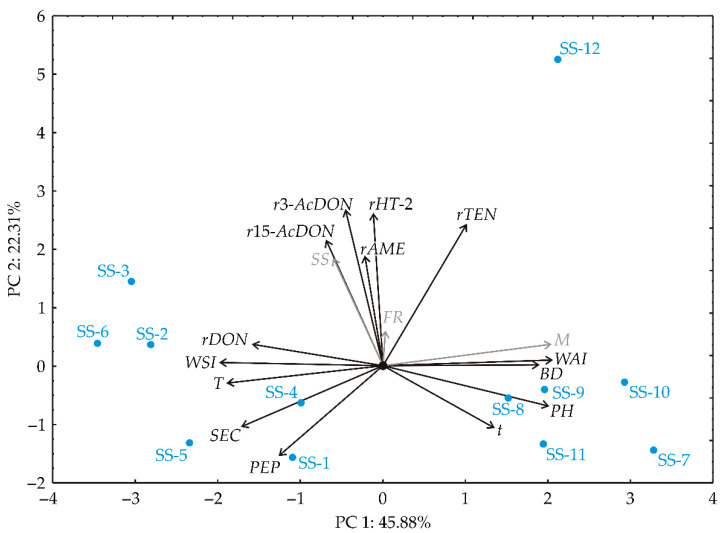
PCA ordination of variables based on component correlations, presented in the first and the second factor plane. *M*—moisture of the material in the extruder barrel (%); *FR*—feeding rate (kg/h); *SS*—screw speed (RPM); *T*—die temperature (°C); *PEP*—pressure at the die (MPa); *SEC*—specific energy consumption (Wh/kg); *t*—mean retention time in the barrel (s); *BD*—bulk density (g/mL); *PH*—pellet hardness (kg); *WAI*—water absorption index (g/g); *WSI*—water solubility index (g/100 g); *rDON*—reduction of deoxynivalenol (%); *r3-AcDON*—reduction of 3-acetyldeoxynivalenol (%); *r15-AcDON*—reduction of 15-acetyldeoxynivalenol (%); *rHT-2*—reduction of HT-2 toxin (%); *rTEN*—reduction of and tentoxin (%); *rAME* (%)—reduction of alternariol monomethyl ether (AME) (%).

When the collected experimental dataset underwent PCA analysis, distinct groupings initially emerged between samples based on the factor variables. As an exploratory tool, this method facilitated the description and differentiation of response variables, as shown in Figure 2. The results of the Principal Component Analysis (PCA) show that the first principal component (PC1) has an eigenvalue of 6.42 and accounts for 45.88% of the total variance. The second principal component (PC2) had an eigenvalue of 3.54 and explained an additional 25.31% of the variance, resulting in a cumulative variance of 71.19% for the first two components. The third component (PC3) contributed 11.06% (eigenvalue = 1.55), while the fourth (PC4) explained 7.67% (eigenvalue = 1.07), resulting in a cumulative variance of 89.92%. Since only the first four components had eigenvalues greater than 1, they are considered significant and together explain about 90% of the total variance. However, as the first two components alone explained more than 70% of the variance, the dimensionality was reduced to two components without significant loss of information. Notably, certain factors, namely *BD*, *PH*, and *WAI,* were more significant in the assessment of the first principal component (PC1) and contributed positively to 11.92%, 13.52% and 14.02%, respectively, on the basis of correlations. In contrast, the variables *T*, *SEC, WSI*, and *rDON* had a negative influence on PC1 and contributed 12.00%, 9.87%, 13.02%, and 8.37% of the total variance, respectively. On the other hand, factors such as *r3-AcDON*, *r15-AcDON*, *rHT-2*, *rTEN*, and *rAME* played more crucial roles in the calculation of the second principal component (PC2) and contributed positively to 21.51%, 13.99%, 20.36%, 17.51%, and 10.70%, respectively. The influence of *PEP* (7.15%) was negative for the calculation of the PC2 coordinate.

The PCA plot in Figure 2 shows clear groupings among the samples, based on moisture content (*M*). The samples with lower moisture content (SS-1–SS-6) are clearly on the left side of the graph. These samples show higher values for temperature (*T*), pressure (*PEP*), specific mechanical energy (*SEC*) and water solubility index (*WSI*) as well as a significant decrease in DON content. In contrast, samples with higher moisture content (SS-7–SS-12) show increased retention time (*t*), pellet hardness (*PH*), bulk density (*BD*), and water absorption index (*WAI*) values. Furthermore, the samples processed at higher screw speeds show a greater reduction in the content of *3-AcDON*, *15-AcDON*, *HT-2*, and *TEN*.

### 3.4. Response Surface Method

The ANOVA analysis was applied to the second-order polynomial models (SOP) to investigate the impacts of the input variables (see Table 2). The results illustrate that in the SOP model for the calculation of temperature (*T*), the linear term of moisture content (*M*) is the most influential variable and has a statistical significance of *p* < 0.001. For the *PEP* evaluation, the SOP model shows the remarkable effect of the linear term of *M* (statistically insignificant at *p* < 0.05). For the *SEC* calculation, the linear term of *M* was statistically significant at the *p* < 0.01 level, while the interchange term of *M × FR* also had a significant impact (statistically insignificant at the *p* < 0.05 level). The linear terms of *SS* were the most influential variable in the SOP model for the *t*-calculation (statistically significant at *p* < 0.001), while *M* and *FR* were also statistically significant factors for the *t*-calculation, at *p* < 0.01 level.

The Pareto charts presented in the Supplement Material Figure S1 provide a visual representation of the standardised effects of different factors (*M*, *FR* and *SS*) and their interactions) on the response variables (*DON*, 3-*AcDON*, 15-*AcDON*, *HT*-2, *AME*, and *TEN* reduction rate, *T*, *PEP*, *SEC*, *t*, *BD*, *PH*, *WAI*, and *WSI*). The charts are based on absolute standardised effect values, which allow for comparison of factor significance.

The SOP model for *BD* calculation was mainly influenced by the linear terms *M* and *FR* (statistically significant at *p* < 0.001 and *p* < 0.01, respectively) and by the interchange terms *M × FR* and *M × SS* (statistically significant at *p* < 0.001 and *p* < 0.01, respectively).

The calculation of the *PH* value was mainly influenced by the linear term of *M* (statistically significant at *p* < 0.001 level). The linear terms of *M, FR* and *SS* were the most influential factors in the SOP model for the *WAI* calculation (statistically significant at *p* < 0.001 level), while the non-linear term of *FR × SS* had an influence on the *WAI* calculation, statistically significant at *p* < 0.01 level).

For the *WSI,* the linear term of M showed significant influence (*p* < 0.001), while the linear term of *SS* reached statistical significance (*p* < 0.05).

Further analysis showed that the linear term of *M*, *FR* and *SS* had a significant effect on the calculation of *rDON* (statistically significant at *p* < 0.001 level), while the interchange term *M × FR* had a statistically insignificant effect on this calculation at *p* < 0.05 level.

Meanwhile, the calculation of *rHT-2* was mainly influenced by the linear term of *SS* in the SOP model, statistically significant at the *p* < 0.01 level. The calculation of *r3-AcDON* was influenced by the linear term of *SS* (statistically insignificant at the *p* < 0.05 level), while the calculation of *rTEN* was mainly influenced by the linear term of *M* in the SOP model (statistically insignificant at the *p* < 0.05 level). The calculation of *r15-AcDON* and *rAME* was not significantly influenced by any term in the SOP models.

All SOP models passed the lack-of-fit tests, ensuring their adequate representation of the data. Furthermore, the high *r*^2^ values confirmed a strong correlation between the model predictions and the experimental results.

**Figure 3:**
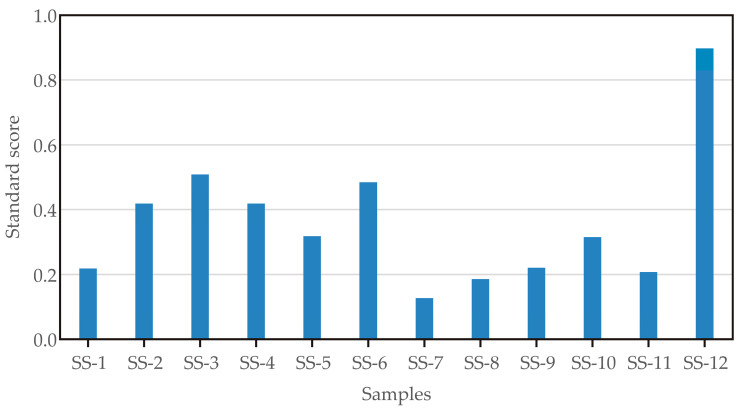
Standard score analysis.

These results are shown in Figure 3. When optimising extruder parameters using standard scores, the optimal score was calculated by averaging the scores across all mycotoxin reduction variables [22]. The function of the maximum score yielded the ideal factor variables and optimal levels for mycotoxin reduction. The highest score was achieved for sample SS-12 (0.897), where the optimised parameters were set as follows: *M* = 24%, *FR* = 25 kg/h, and *SS* = 480 RPM. Under these conditions, the extrusion process yielded *T* = 103.5 °C, *PEP* = 1.0 MPa, *SEC* = 91.5 Wh/kg, *t* = 24.0 s. In addition, the physicochemical properties of the optimum sample were determined: *BD* = 0.352 g/mL, *PH* = 13.7 kg, *WAI* = 8.96 g/g, and *WSI* = 14.9 g/100 g (see Table 1). At these optimum extrusion conditions, the reduction rates of the examined mycotoxins were as follows: 3.8% for DON, 60.7% for 3-AcDON, 61.5% for 15-AcDON, 86.5% for HT-2, 47.7% for TEN, and 55.9% for AME.

Sample SS-3 (Figure 3) followed the optimum result and achieved a score of 0.508. Sample SS-3 was produced with the extruder parameters *M* = 20%, *FR* = 20 kg/h, and *SS* = 480 RPM. Under these conditions, the extrusion process yielded *T* = 134.6 °C, *PEP* = 1.5 MPa, *SEC* = 143.0 Wh/kg, *t* = 16.0 s. In addition, the physicochemical properties of sample S-3 were determined: *BD* = 0.186 g/mL, *PH* = 9.08 kg, *WAI* = 4.66 g/g, and *WSI* = 35.4 g/100 g (see Table 1). At these extrusion conditions, the reduction rates of the analysed mycotoxins were as follows: 4.49% for DON, 18.0% for 3-AcDON, 56.3% for 15-AcDON, 54.1% for HT-2, 6.28% for TEN, and 43.3% for AME.

It should be noted that sample SS-3 showed better quality characteristics of its pellets (BD, PH and WSI), but the reduction rate of analysed mycotoxins is lower (with the exception of DON) is lower than in sample SS-12.

## 4. Discussion

### 4.1. Mitigation of Mycotoxins During Extrusion Processing

Investigations concerning the fate of other, mainly unregulated mycotoxins during the extrusion process of cereals are limited and mainly refer to the studies published by Janić Hajnal et al. [21,22,23]. The results obtained in this study can be compared with the results of the earlier study by Janić Hajnal et al. [22], as the same raw material but a different type of extruder was used for extrusion. The extrusion process parameters of a co-rotating twin-screw extruder with the medium screw speed (650 RPM), the highest feeding rate (30 kg/h), and the lowest moisture content of the raw material (20%) yielded the best standard score (0.73) and provided the best reduction rates of the mycotoxins present (9.5, 27.8, 28.4, 60.5, 12.3 and 85.7% for DON, 3-AcDON, 15-AcDON, HT-2, TEN and AME, respectively) in the final product [22]. In the present study, the optimal process parameters of the single-screw extruder, which provide the best standard score (0.897) for mycotoxin reduction (3.80, 60.7, 61.5, 86.5, 47.7, and 55.9% for DON, 3-AcDON, 15-AcDON, HT-2, TEN and AME, respectively) were achieved at the highest screw speed (480 RPM), the highest feeding rate (25 kg/h) and the highest moisture content of the raw material (24%). As can be seen, with the optimum process parameters for both extruders, a greater reduction for 3-AcDON, 15-AcDON, HT-2, and TEN was achieved by using the single-screw extruder, while a greater reduction of DON and AME content was achieved by using a twin-screw extruder.

Most of the published findings on the ability of the extrusion process to reduce the concentration of *Fusarium* toxins in cereals relate to the reduction of DON in wheat [37]. The research results published so far show contradictory findings on the effects of extrusion processing on DON reduction. According to the published data reviewed by Schaarschmidt and Fauhl-Hassek [37], the DON concentration of whole grain wheat flour during the extrusion process varied between +1 and −23% (twin-screw extruder), while the DON reduction rate during the extrusion process (twin-screw extruder) of soaked wheat grains varied between 6 and 10%. The results of this study are consistent with the above-mentioned reduction rate of DON during extrusion, regardless of whether a twin-screw or a single-screw extruder was used, as similar influences of the process parameters led to maximum reductions of 7.27% (SS-6) and 16.6% [22]. The interchange term of moisture content of the material in the extruder barrel and feeding rate had a statistically minor impact on the calculation of DON reduction at the *p* < 0.05, while the linear terms of the moisture content of the material in the extruder barrel, feeding rate and screw speed had a considerable impact on the calculation of the DON reduction (statistically significant at the level *p* < 0.001). However, the extrusion of whole-grain triticale flour via a twin-screw extruder using the experimental design conducted by Janić Hajnal et al. [22] resulted in a higher DON reduction (0.12–16.5%) compared to the present study (0.72–7.27%), although DON was not significantly affected by any term in the SOP models. Wu et al. [38] investigated how various physicochemical factors, including barrel temperature (100–170 °C), moisture content (17 and 34%), a compression ratio of a screw (1:1 and 3:1), residence time (2 and 4 min), pH (6.0, 7.3, 7.8 and 8.0) and protein content (10 and 20% gluten addition) influenced the reduction of DON levels in fortified wheat (at a level of 500 µg/kg) during the extrusion process in a laboratory-scale conical single-screw extruder. The DON reduction rates varied between 3.1% and 79.2% depending on the variation of the applied physicochemical factors. Wu et al. [38] emphasised that the reduction rates of DON increased with increasing barrel temperature, higher screw compression at a barrel temperature below 160 °C and lower screw speed. According to Pleadin et al. [39], the extrusion of maize, wheat, and oats using a single-screw laboratory extruder effectively reduces the DON content in the range of 51% to 87%, depending on the type of cereal used and the extruder temperature regime applied of the at constant screw speed (100 RPM), dosing speed (40 RPM), screw compression ratio (4:1), and raw material moisture content (25%). The maximum reduction rate of DON content of 87%, 71% and 66% in oats, wheat and maize, respectively, was achieved when a temperature profile of 135–190–190 °C (dosing/compression/ejection zone) of the extruder was used [39]. In the study by Srečec et al. [40], the DON content in maize extrudates was reduced by 60.29% after extrusion treatment of maize (after 7 months of storage) at 130 °C and 35 bar for 30 s was used. Furthermore, in the study by Cazzaniga et al. [41], the DON content (5 mg/kg) in red maize flour (moisture 15% and 30%) with or without additive (sodium metabisulphite addition (1%)) was reduced by more than 95% during extrusion processing via a laboratory-scale extruder at a temperature of 150 °C and 180 °C, a screw compression ratio of 3:1 and a screw speed of 120 RPM. Differences in DON reduction may be caused by variations in temperature, moisture content, screw compression level, and additives [38]. In addition, in 2017, Wu et al. [42] investigated the fate of DON and deoxynivalenol-3-glucoside during cereal-based thermal food processing. In relation to the extrusion process and based on the available data, the authors concluded that higher moisture content and screw compression lead to significantly higher DON reduction in cereal samples, i.e., the two main variables influencing DON reduction during the extrusion process are the moisture content of the raw material and the compression rate [42].

Considering the fate of 3-AcDON and 15-AcDON, it should be noted that a higher reduction rate (0.26–60.7% and 7.13–66.7%, respectively) was achieved for both mycotoxins in the present study compared to the reduction ranges achieved by using a twin-screw extruder (1.70–32.80% and 1.70–45.7%, respectively) [22]. In the current investigation, the linear term of the screw speed influenced the calculation of the reduction of 3-AcDON (statistically significant at the *p* < 0.05 level), while the reduction of 15-AcDON was not significantly affected by any term in the SOP models. For extrusion processing of whole grain triticale flour via a twin-screw extruder, the quadratic terms of moisture content were the most effective for calculating 3-AcDON reduction in the SOP models (statistically significant at the *p* < 0.05 level), and again the reduction of 15-AcDON was not significantly affected by any term in the SOP models [22].

A comparable behavioural pattern was observed for HT-2 toxin. In particular, lower reduction rates (24.26–60.46%) were observed in the applied experimental design for the extrusion of whole-grain triticale flour on a twin-screw extruder, compared to the results of the current study (17.3–86.5%). When calculating the reduction of HT-2 toxin in SOP models, the quadratic terms of moisture content were most effective in the extrusion of whole-grain triticale flour (statistically significant at *p* < 0.05) [22], while in the current study, the linear term of screw speed in the SOP model had the most influence, statistically significant at *p* < 0.01. In the study conducted by Pleadin et al. [43], the extrusion of maize, triticale and wheat flour with a single-screw laboratory extruder effectively reduced T-2/HT-2 toxins levels from 73.0 to 92.5% depending on the cereal used and extruder temperature regime applied at constant screw speed (100 RPM), dosing speed (40 RPM), and moisture content of the raw material (25%). The authors emphasised that among the thermal processes, the extrusion process appears to be the most efficient, as it led to the almost complete elimination of T-2/HT-2 toxins in investigated cereal grains, regardless of the temperature regime used during extrusion [43]. In particular, when triticale flour was extruded at three different temperature regimes, the reduction of T-2/HT-2 toxins ranged from 82.8 to 85.9%. In another study, the degradation of T-2 and HT-2 toxins during laboratory and industrial extrusion processing of oats was systematically examined by Schmidt et al. [44]. When oats fortified with T-2 or HT-2 toxins (500 μg/kg each) were extruded on a laboratory single-screw extruder, the reduction rates of T-2 and HT-2 toxins were 59.6 ± 1.51% and 47.2 ± 0.53%, respectively. On the other hand, when naturally contaminated oat flour dust was extruded under laboratory conditions, the reduction rates of T-2 and HT-2 toxins were lower (35.1 ± 1.55% and 22.0 ± 4.68%, respectively). Furthermore, during extrusion under industrial conditions (twin-screw extruder) of a mixture that contained naturally contaminated oat flour, the reduction rate of T-2 and HT-2 toxins was 19.5 ± 6.28% and 11.7 ± 7.70%, respectively. The authors emphasised by optimising the process parameters (temperature, mechanical shear, and moisture content) that influence the degradation of these mycotoxins, safe food can be produced [44].

Regarding the fate of TEN during extrusion processing, the TEN reduction rate varied from 1.7% to 21.2% when whole-grain triticale flour was extruded via a twin-screw extruder [22]. It was primarily influenced by the linear terms of screw speed and moisture content, as well as the quadratic term of screw speed and the non-linear term of screw speed and feeding rate in the SOP model (*p* < 0.01) [22]. The linear terms of feeding rate and the non-linear term of the quadratic term of screw speed and moisture content significantly contributed (at the *p* < 0.05 level) to the calculation of TEN reductions. However, in the present study, a higher reduction rate of 1.77% to 47.7% was obtained for TEN, which was mainly influenced by the linear term of the moisture content of the material in the extruder barrel in the SOP model (statistically significant at *p* < 0.05). It is worth mentioning that higher TEN reduction rates (43.1–56.7%) were also achieved when whole-grain sorghum flour was extruded using a twin-screw [23]. With the input parameters of a twin-screw extruder used, the reduction rate of TEN was significantly influenced by the linear term of the moisture content of the material in the extruder barrel in the SOP model (statistically significant at the *p* < 0.05 level) [23].

Similar to DON, a higher reduction of AME was achieved in the study by Janić Hajnal et al. [22]. In particular, the reduction rate of AME in whole-grain triticale flour extruded with a twin-screw extruder varied from 53.2% to 91.8%, and it was mainly influenced by the linear term of the screw speed in the SOP model calculation (*p* < 0.05) [22]. In the present study, its reduction rate varied from 10.6% to 55.9%, when using a single-screw extruder and it was not significantly affected by any term in the SOP models. On the other hand, when whole-grain wheat flour was extruded using a single-screw extruder, the AME reduction rate varied from 62.8% to 94.5%, and the calculation of AME reduction was influenced by the linear term of moisture content and the quadratic term of screw speed in the SOP models (statistically insignificant, at the *p* < 0.10 level) [21]. Furthermore, during the extrusion process of whole-grain sorghum flour via a twin-screw extruder, its reduction rate varied from 71.1% to 80.0% [23]. Concerning the influence of processing parameters on the reduction rate of AME, it was not significantly affected by the linear terms of screw speed and moisture content of the material in the extruder barrel (*p* > 0.05 level) [23].

### 4.2. Effects of Extrusion Condition on Quality Indicators of Triticale Pellets

Concerning the quality indicators for triticale pellets, the bulk density (*BD*) of the pellets produced in the present study under optimal extrusion conditions is lower (0.352 g/mL) than the bulk density (0.589 g/mL) obtained with a pilot twin-screw extruder at optimal process parameters [22], resulting in pellets with a higher degree of expansion were obtained. The low density is a favourable attribute of the extruded product. In the study conducted by Doğan, and Karwe [45], RSM was used to analyse the effects of temperature (130–170 °C), screw speed (250–500 RPM), and moisture content of the material in the extruder barrel (16–24%) at a fixed feeding rate of 300 g/min of a twin-screw extruder on the physicochemical properties of quinoa extrudates. The response surface shows that the moisture content of the raw material in the extruder barrel significantly affects the bulk density, followed by the screw speed, while the temperature seems to have a negligible effect [45]. In the present study, the linear terms of moisture content (*M*) and feeding rate (*FR*) (statistically significant at *p* < 0.001 and *p* < 0.01 level, respectively) and interchange terms *M* × *FR* and *M* × *SS* (statistically significant at *p* < 0.001 and *p* < 0.01 level, respectively) had a substantial impact on the SOP model for *BD* computation. In the case of triticale extrusion with a twin-screw extruder with experimental design applied, the *BD* was altered by the non-linear term of screw speed and (*p* < 0.05 level) in the SOP model [22].

Hardness is the maximum force required to break the extrudate and is related to the expansion and cell structure of extrudates [46]. A high moisture content of the material leads to a higher density of the extrudate and thus to a higher hardness, while a high screw speed and a high temperature can lower melt viscosity, reducing the bulk density and hardness of the extrudates [47]. In the current study, the recorded pellet hardness (*PH*) at optimal process parameters is lower (13.7 kg) compared to the *PH* (19.4 kg) achieved with a pilot twin-screw extruder [22]. The moisture content of the material in the extruder barrel had the greatest influence on hardness (*p* < 0.001), while for triticale pellets produced with a twin-screw extruder, hardness was also influenced by the linear term of the moisture of the material in the extruder barrel (*p* < 0.01). Additionally, in the SOP models, it was influenced by the non-linear term of the screw speed and the moisture of the material in the extruder barrel (*p* < 0.05) [22]. Similar patterns were found for expanded snacks made from rice [48], wheat [49], chickpea flour [50], expanded intermediate wheatgrass products [47], and sorghum [23].

The *WAI* and *WSI* indicate the ability of an extruded product to interact with water [51]. Depending on the presence of hydrophilic groups, which regulate the binding of water molecules, the *WAI* measures the quantity of water absorbed by the starch and serves as a marker for gelatinisation. The *WSI* indicates the degree of starch conversion during extrusion, which is ascribed to gelatinisation and dextrinisation, and measures the soluble components released from the starch (soluble polysaccharide) and serves as a measure of molecular component degradation [48]. The conditions of extrusion processing influence the *WAI* and *WSI* of extrudates. A high moisture content of the material in the extruder barrel reduces shear and starch degradation, while a low temperature restricts water availability. Extrusion at low moisture and high screw speed leads to low *WAI* and high *WSI* [52]. Ding et al. [48] investigated the effects of extrusion conditions on the physicochemical properties of rice-based extrudates. They found that increasing the feeding rate results in extrudates with lower *WSI* while increasing the moisture content of the material in the extruder barrel results in extrudates with higher *WAI* and lower *WSI* values. They also emphasised that higher barrel temperatures increased the *WSI* of the extrudate, while *WAI* and *WSI* of the extrudate were not significantly affected by the screw speed. In another study from 2006, the same authors investigated the effects of extrusion conditions on the functional and physical properties of expanded wheat-based snacks [49]. Based on their results, they concluded that increasing the feeding rate leads to extrudates with higher expansion, lower *WSI* and higher hardness. The results of the current study indicate that increasing the moisture content of the material in the extruder barrel leads to an increase in the *WAI* and a decrease in the *WSI* of extrudates (Table 1) as water acts as a plasticiser, possibly reducing shear and starch degradation during extrusion [53]. On the other hand, an increase in screw speed leads to a decrease in *WAI* and an increase in *WSI* [52]. Consequently, a low *WAI* and a high *WSI* are achieved when the material is extruded at a high screw speed and a low moisture content of the material in the extruder barrel [52,53]. The results in this study show the same dependence on the screw speed, the moisture content of the material in the extruder barrel and the die temperature and their influence on the *WAI* and *WSI* of extrudates (Table 1).

In addition, the present study showed better results for *WAI* (8.98 g/g) and *WSI* (35.5 g/100 g) under optimal extrusion conditions compared to the extrusion of whole-grain triticale flour using a twin-screw extruder (*WAI* = 4.96 g/g; *WSI* = 9.1 g/100 g) [22]. In the SOP model of the present study, the linear terms of the moisture content of the material in the extruder barrel, the feeding rate, and the screw speed had the greatest influence on the calculation of *WAI* (statistically significant at the *p* < 0.001 level). The non-linear term of feeding rate and screw speed also had an impact on the calculation of the *WAI* (statistically significant at the *p* < 0.01 level). The linear term of the moisture content of the material in the extruder barrel showed a significant influence on the *WSI* at the *p* < 0.001 level, while the linear term of the screw speed reached statistical significance at *p* < 0.05. In the extrusion of whole grain triticale flour via a twin-screw extruder, the *WAI* and *WSI* were influenced by the quadratic term of the screw speed, while the *WAI* was also influenced by the linear term of the screw speed (statistically significant at the *p* < 0.01 level) [22].

## 5. Conclusions

In brief, this study has shown that extrusion processing with a single-screw extruder is an effective and promising method for reducing the levels of investigated *Fusarium* and *Alternaria* toxins in food and feed items. This process enables the food and feed sector to offer safe and healthy products while mitigating the negative effects of mycotoxins on health. The optimal parameters of the extrusion process, with the highest moisture content of the raw material in the extruder barrel (*M* = 24%), the highest feeding rate (*FR* = 25 kg/h), and screw speed (*SS* = 480 RPM) achieved the best standard scores (0.897) and provided the most effective reduction rates of mycotoxins present in the final product. Future research should focus on investigations that provide an understanding of the chemical changes associated with the fate of *Fusarium* and *Alternaria* toxins and the toxicity of their degradation products during food processing to prevent the potential hazards associated with processed grains.

## Figures and Tables

**Table 1:** Technological parameters of extrusion, reduction of mycotoxins, and quality indicators of pellets.

Sample	*M*	*FR*	*SS*	*T*	*PEP*	*SEC*	*t*	*BD*	*PH*	*WAI*	*WSI*	*rDON*	*r*3-*AcDO*N	*r*15-*AcDO*N	*rHT*-2	*rTEN*	*rAME*
SS-1	20	20	300	128.5	0.42	140.8	25.0	0.240	11.1	6.66	21.5	3.28	12.1	27.1	19.7	3.06	25.5
SS-2	20	20	390	141	0.31	134.2	22.0	0.207	9.56	5.50	35.5	3.85	14.2	66.7	47.9	1.77	27.0
SS-3	20	20	480	134.6	0.55	143.0	16.0	0.186	9.08	4.66	35.4	4.49	18.0	56.3	54.1	6.28	43.3
SS-4	20	25	300	130.4	0.26	133.8	31.0	0.231	13.2	6.44	23.9	6.38	8.9	36.3	17.3	3.96	54.4
SS-5	20	25	390	132	0.41	186.6	23.0	0.234	11.4	6.50	24.9	6.41	17.8	26.2	38.2	4.96	13.1
SS-6	20	25	480	141	0.28	186.1	20.0	0.183	9.13	5.56	33.2	7.27	27.2	19.6	57.8	15.9	26.8
SS-7	24	20	300	103.8	0.20	116.6	35.0	0.396	17.7	8.66	16.2	0.72	1.9	17.9	31.0	15.3	13.2
SS-8	24	20	390	134	0.11	128.7	26.0	0.401	14.5	7.84	19.7	1.20	0.3	22.8	52.4	13.2	11.7
SS-9	24	20	480	113.6	0.21	117.7	18.0	0.418	18.4	7.72	19.0	1.99	4.5	24.8	45.0	18.4	10.6
SS-10	24	25	300	97.6	0.10	110.9	34.0	0.298	16.9	8.96	12.6	3.22	1.0	34.0	26.6	18.1	35.6
SS-11	24	25	390	100.1	0.23	113.5	30.0	0.301	16.8	8.98	20.5	3.69	2.2	7.13	35.5	2.06	32.9
SS-12	24	25	480	103.5	0.10	91.5	24.0	0.352	13.7	8.96	14.9	3.80	60.7	61.5	86.5	47.7	55.9
*p*-value				*	*	*	*	*	*	*	*	*	*	*	*	*	*

* Statistically significant, at *p* < 0.05 level. *M*—moisture of the material in the extruder barrel (%); *FR*—feeding rate (kg/h); *SS*—screw speed (RPM); *T*—die temperature (°C); *PEP*—pressure at the die (MPa); *SEC*—specific energy consumption (Wh/kg); *t*—mean retention time in the barrel (s); *BD*—bulk density (g/mL); *PH*—pellet hardness (kg); *WAI*—water absorption index (g/g); *WSI*—water solubility index (g/100 g); *rDON*—reduction of deoxynivalenol (%); *r3-AcDON*—reduction of 3-acetyl deoxynivalenol (%); *r15-AcDON*—reduction of 15-acetyl deoxynivalenol (%); *rHT-2*—reduction of HT-2 toxin (%); *rTEN*—reduction of and tentoxin (%); *rAME* (%)—reduction of alternariol monomethyl ether (AME) (%).

**Table 2:** ANOVA evaluation of technological parameters and reduction of mycotoxins (sum of squares).

	df	*T*	*PEP*	*SEC*	*t*	*BD*	*PH*	*WAI*	*WSI*	*rDON*	*r*3-*AcDON*	*r*15-*AcDON*	*rHT*-2	*rTEN*	*rAME*
*M*	1	1999.501 ***	6.453 *	5023.339 **	75.000 **	0.065 ***	99.175 ***	20.784 ***	423.888 ***	24.253 ***	62.921	339.658	145.416	517.010 *	76.104
*FR*	1	215.901	0.003	142.554	33.333 **	0.005 **	0.054	1.592 ***	24.981	19.350 ***	370.997	79.103	11.532	100.412	638.615
*SS*	1	131.220	0.720	164.711	276.125 ***	0.000	9.172	1.824 ***	101.355 *	1.942 ***	935.810 *	276.496	2768.497 **	287.800	7.805
*SS* ^2^	1	156.060	0.667	305.164	0.042	0.000	0.876	0.000	25.029	0.031	177.365	42.638	4.222	299.809	384.139
*M × FR*	1	204.188	0.213	1530.473 *	0.333	0.006 ***	4.199	0.081	1.044	0.224 *	189.705	923.268	66.793	4.354	680.408
*M × SS*	1	0.125	0.845	662.844	6.125	0.004 **	1.594	0.473	40.761	0.008	180.485	60.149	0.122	38.625	93.503
*FR × SS*	1	0.045	0.980	110.261	3.125	0.000	4.489	0.538 **	3.279	0.130	603.180	80.702	337.581	155.592	63.264
Error	4	348.283	3.535	1020.300	16.583	0.001	9.729	0.269	52.598	0.136	626.060	2031.767	709.390	404.596	851.539
R^2^		0.886	0.737	0.886	0.960	0.988	0.925	0.989	0.922	0.997	0.801	0.470	0.825	0.776	0.695
adjR^2^		0.687	0.275	0.687	0.889	0.967	0.793	0.971	0.785	0.992	0.453	0.000	0.518	0.385	0.162

*** Statistically significant at *p* < 0.001 level, ** statistically significant at *p* < 0.01 level, * statistically significant at *p* < 0.05 level. *M*—moisture of the material in the extruder barrel (%); *FR*—feeding rate (kg/h); *SS*—screw speed (RPM); *T*—die temperature (°C); *PEP*—pressure at the die (MPa); *SEC*—specific energy consumption (Wh/kg); *t*—mean retention time in the barrel (s); *BD*—bulk density (g/mL); *PH*—pellet hardness (kg); *WAI*—water absorption index (g/g); *WSI*—water solubility index (g/100 g); *rDON*—reduction of deoxynivalenol (%); *r*3-*AcDON*—reduction of 3-acetyl deoxynivalenol (%); *r*15-*AcDON*—reduction of 15-acetyl deoxynivalenol (%); *rHT*-2—reduction of HT-2 toxin (%); *rTEN*—reduction of and tentoxin (%); *rAME* (%)—reduction of alternariol monomethyl ether (AME) (%).

## Data Availability

The original contributions presented in the study are included in the article/Supplementary Material, further inquiries can be directed to the corresponding author.

## Supplementary Materials

The following supporting information can be downloaded at: https://www.mdpi.com/article/10.3390/foods14020263/s1, Figure S1: Pareto Chart of Standardized Effects, for (a) *T*, (b) *PEP*, (c) *SEC*, (d) *t*, (e) *BD*, (f) *PH*, (g) *WAI*, (h) *WSI*, (i) *rDON*, (j) *r*3-*AcDON*, (k) *r*15-*AcDON*, (l) *rHT*-2, (m) *rTEN*, (n) *rAME*.
