# Supplementary figures and images for: Mitigation of Mycotoxin Content by a Single-Screw Extruder in Triticale (x Triticosecale Wittmack)
Source: Foods. 2025 Jan 15;14(2):263. doi: 10.3390/foods14020263 (PMC11765161; doi:10.3390/foods14020263)

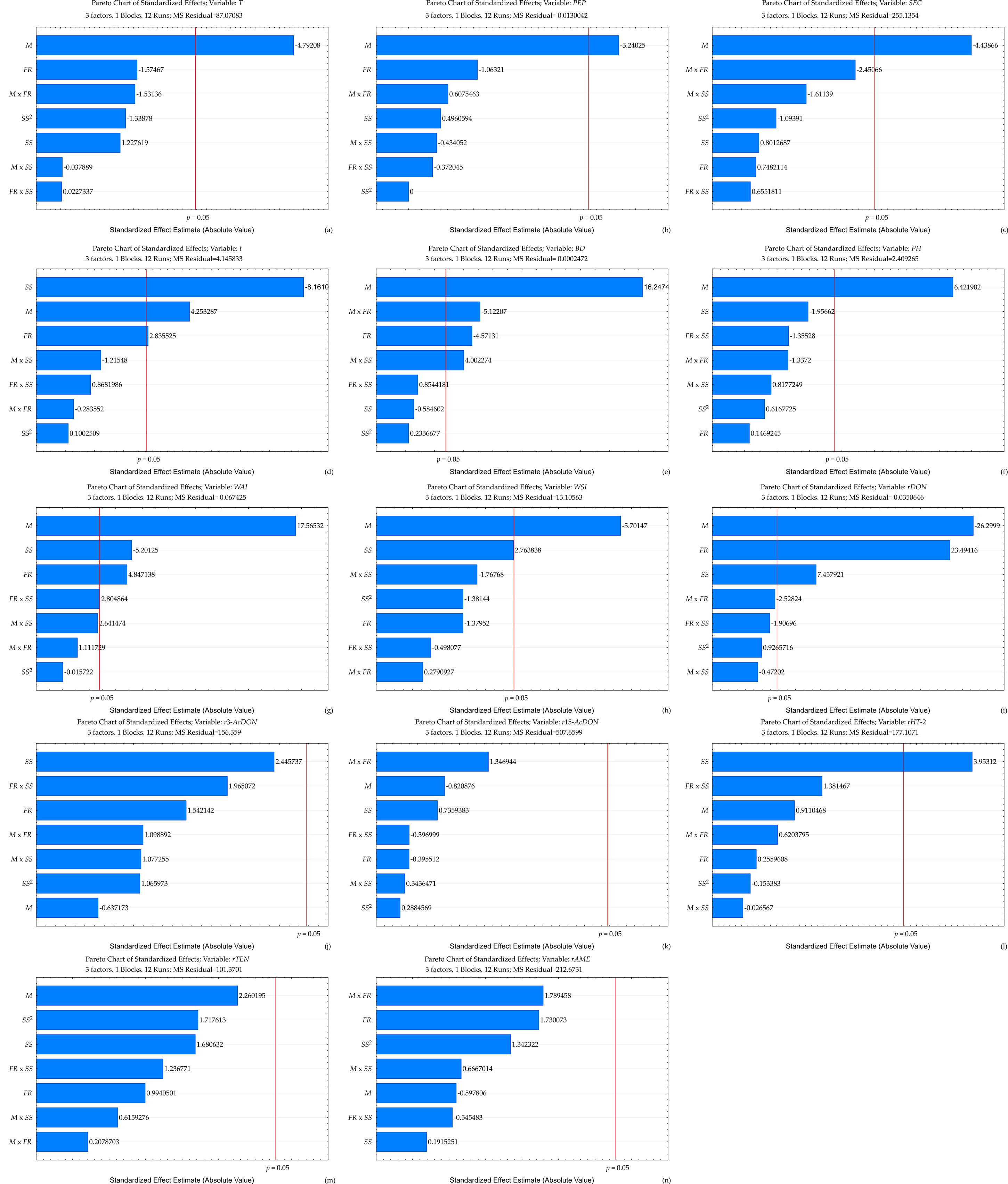

Supplement: Supplementary file 1 [file foods-14-00263-s001.zip › foods-3377765-supplementary.tif]
